# A Tough Case to Crack: Diagnostic, Ethical, and Legal Considerations in Treating Compulsive Neck Cracking

**DOI:** 10.7759/cureus.23875

**Published:** 2022-04-06

**Authors:** Andrea Johnson, Alexander Linse, Kenneth C Novoa

**Affiliations:** 1 Psychiatry, University of Colorado School of Medicine, Aurora, USA; 2 Psychiatry, Denver Health Medical Center, Denver, USA

**Keywords:** ethics, autonomy, beneficence, pain, cracking, popping, neck, anxiety, somatic symptom disorder, ocd

## Abstract

Compulsive behaviors rarely lead to significant physical injury, but when they do, they can introduce challenges in treatment secondary to diagnostic uncertainty and introduce ethical and legal dilemmas when trying to optimize patient care. We discuss the clinical complexities in treating a patient with compulsive neck cracking as she navigates various clinical settings in hopes of alleviating the anxiety and pain that lead to her behaviors. Ultimately, the principles of beneficence and autonomy must be weighed when determining whether someone with a chronic risk of serious physical harm from compulsive behaviors requires involuntary psychiatric treatment.

## Introduction

Obsessive-compulsive disorder (OCD) is a psychiatric illness that can have a significant negative impact on an individual’s health and well-being. It is now recognized that the lifetime prevalence rate of obsessive-compulsive disorder is around 2.3% of the population in the United States [1]. The mean age of onset is 19.5 years, and the onset of symptoms is typically gradual but can also be acute [1]. The Diagnostic and Statistical Manual of Mental Disorders, Fifth Edition (DSM-5) classifies obsessive-compulsive disorder as the presence of obsessions, compulsions, or both. Obsessions are characterized as thoughts, images, or urges that are both persistent and recurrent and are experienced as intrusive and unwanted. Often, these can cause marked anxiety or distress. The affected persons attempt to curtail or nullify these obsessions via another thought or action (i.e., compulsions). The compulsive component of OCD is a set of repetitive physical or mental acts that the individual undergoes in response to an obsession. They must be aimed at reducing anxiety and distress and are enacted in a manner that is excessive and often not realistically related to the obsession in which they are aimed [2].

Although compulsive behaviors may result in minor physical injuries, more significant injuries are typically considered rare [3]. A review of the literature reveals that these uncommon major complications may include genital mutilation, loss of vision, severe infection, or rectal prolapse [4-7]. There has also been one report of tibial stress fracture related to compulsive jump roping, as well as a single case report of compulsive joint clicking [3,8].

Here, we present the case of a patient who experienced a cerebrovascular accident due to compulsive cracking of their cervical spine. The patient’s symptoms both in characterization and chronology made diagnostic clarity difficult. This, coupled with the ego-dystonic nature of her physically dangerous compulsive behavior and her consistent denial of self-harm motivation, can present treatment teams with ethical and legal decisions to make when considering how to proceed with future treatment. The patient gave us informed consent for the publication of this case report as per the 2017 Case Report (CARE) guidelines [9].

## Case presentation

Ms. M is a 19-year-old female with a history of “mild anxiety” who presented to the emergency department (ED) for chronic neck pain of approximately 18 months duration. The pain started in her neck and over time began to migrate to other joints as well (e.g., started to pop her toes), leading to multiple ED visits and specialty referrals. She was diagnosed with a Baker’s cyst on ultrasound, which was believed to be secondary to the patient popping her knees excessively. Approximately four months prior to this encounter, the patient started to experience pain in her neck again, which is when she started to compulsively pop her neck to try and relieve this sensation. The compulsive behavior was so frequent that she presented to an ED with extensive bilateral conjunctival hemorrhages and bruising under her eyes attributed to repeated pressure from her neck manipulation and popping. At that time, computed tomography angiography (CTA), cervical spine X-ray, and magnetic resonance imaging (MRI) of the brain were all normal (no CT of the cervical spine was done). She was prescribed medication for the pain and advised to follow up with outpatient rheumatology and behavioral health services to address anxiety.

**Figure 1:**
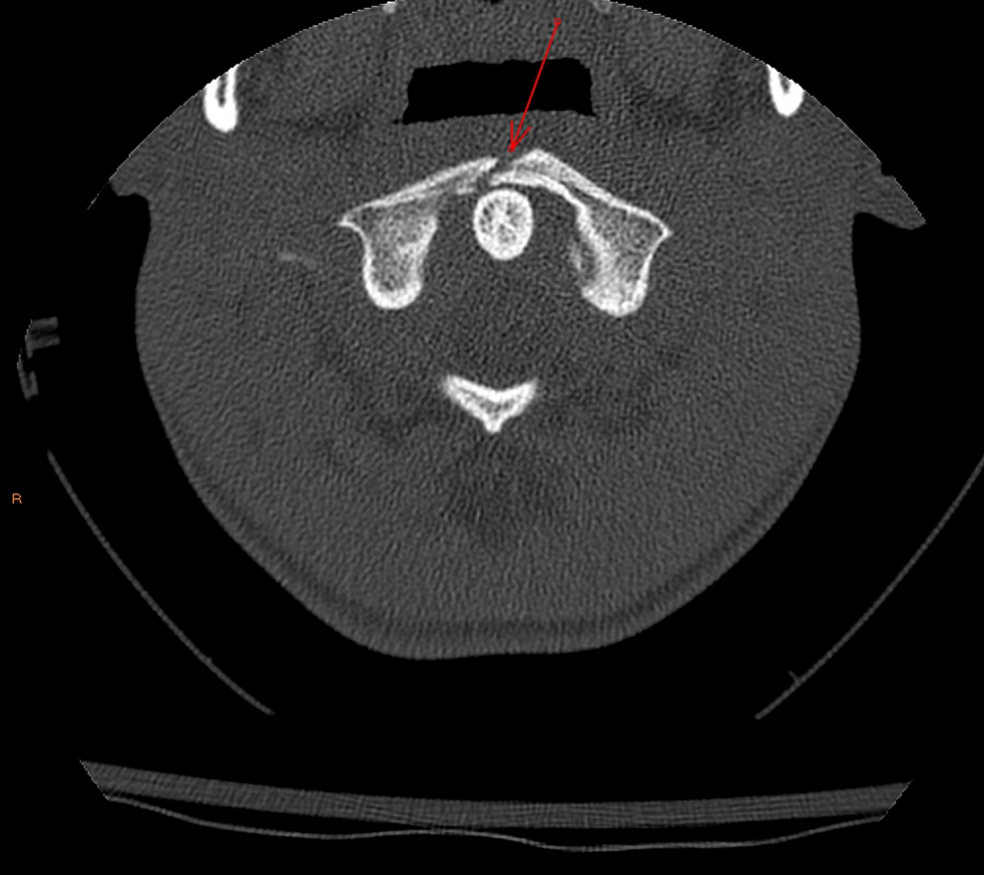
C1 burst fracture (anterior) - axial image

**Figure 2:**
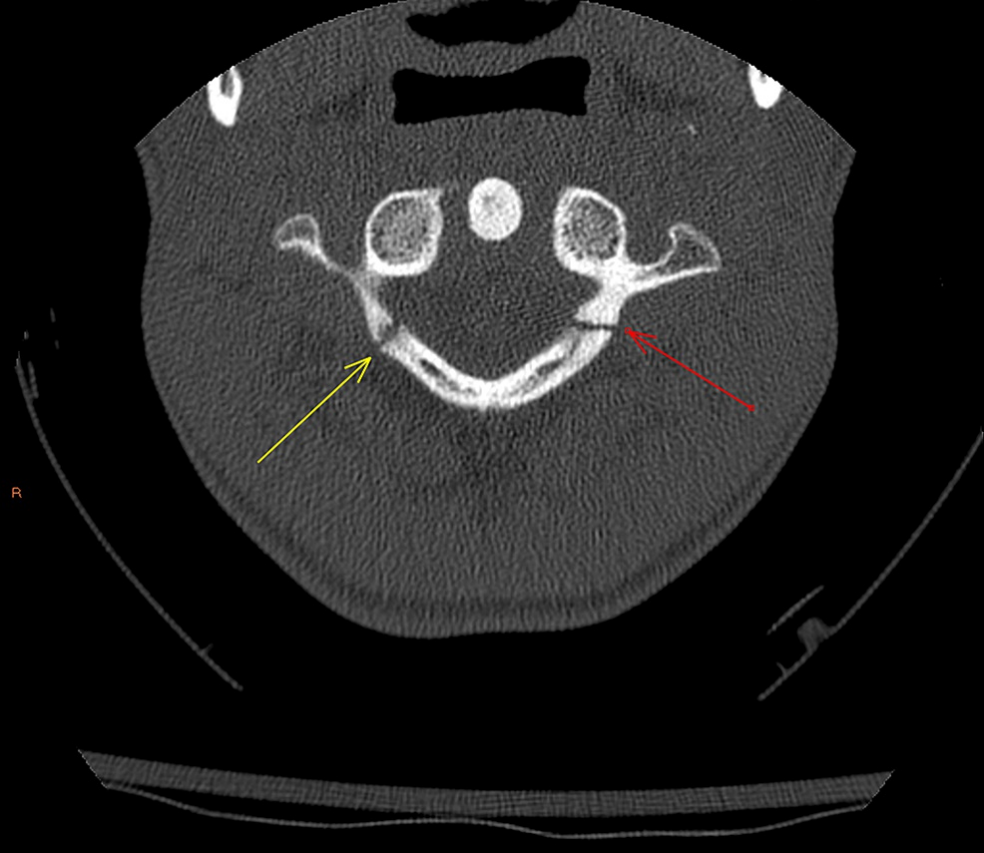
C1 burst fracture (posterior) - axial image

During this encounter, Ms. M reported that the pain she feels in her neck leads her to feel anxious and as though she must alleviate this pain by popping her neck. She reported engaging in this behavior more than 20 times a day. Spinal X-ray and CT of the cervical spine now showed a burst-type fracture of the C1 vertebral body (Figures 1-2). This was not seen in imaging done four months prior, but there was speculation that this may have been an old injury from cheerleading that was missed on prior imaging (no CT cervical spine was done then). She was placed in a C-collar and evaluated by neurosurgery, who recommended admission to the pediatric intensive care unit for monitoring. Psychiatry was then consulted for evaluation of possible compulsive behaviors and anxiety.

Information gathered from the patient and her mother revealed further details about her history with anxiety. Ms. M would have repetitive behaviors as a child where she would chew on her shirt and nails. As she got older, she would bite her fingernails and her lips repetitively in response to anxiety. When the pain in her joints started to migrate across her body in the last 18 months, Ms. M also started to compulsively pick at her face, leaving noticeable marks that she was always hesitant to talk about. She spoke about how the popping of her neck would result in only a temporary (~20 minutes) physical and emotional relief from her pain and anxiety, before feeling compelled to repeat this behavior. At the time of our first evaluation with her, she reported being able to stop herself due to fear of the potential consequences of a worsened neck injury. She was able to explain that she understood the potential adverse physical outcomes of continuing this behavior (e.g., paralysis and stroke), denied suicidal ideation, and denied any intent of self-harm through these compulsive behaviors.

Given the nature of Ms. M’s symptoms, we performed a Yale-Brown Obsessive-Compulsive Scale (Y-BOCS) screening. She scored 28 out of 40, indicating moderate-severe OCD symptoms, primarily involving overwhelming, intrusive, obsessive thoughts and compulsions related to popping joints. To address anxiety, she tried hydroxyzine with little benefit and limited trials of escitalopram and propranolol with no noted benefit. She reported using cannabis (concentrates) multiple times per day with increased use over the prior several months to address anxiety. She denied alcohol and illicit drug use.

With OCD high on the differential, the consultation-liaison (C-L) psychiatry team started Ms. M on fluvoxamine and recommended voluntary inpatient psychiatric admission given the risk of significant harm with continued neck popping behaviors and her limited ability to control these compulsions in the context of anxiety and pain. Upon psychiatric admission that day, Ms. M quickly changed her mind and asked to be released. Without obtaining first-hand collateral themselves and given the severity of her injury, they placed her on a 72-hour involuntary hold for grave disability. After speaking to her mother and speaking more in depth with Ms. M, the psychiatric providers felt that her presentation may be more due to uncontrolled anxiety and somatic symptom disorder and switched fluvoxamine to fluoxetine. After ~18 hours in the psychiatric unit, the patient maintained her cervical neck collar and did not try to pop her neck, and the providers felt that she no longer met the criteria for an involuntary hold. Ms. M was discharged with instruction to continue fluoxetine 10 mg daily and follow up with a community mental health center and neurosurgery clinic.

Approximately five weeks after her discharge, Ms. M again presented to the ED with worsened neck pain as well as new weakness and numbness of her right arm. Her mother had raised concerns about “suicidal thoughts” based on reported texts she received from the patient, but Ms. M on evaluation was denying any active suicidal thoughts. When told that she would have to see the psychiatrist in the ED for safety evaluation, Ms. M became frustrated and said she would go home and continue to pop her neck. At this time, the patient was placed on a 72-hour involuntary hold for danger to self and admitted to pediatric medicine for assessment and treatment of neck pain. C-L psychiatry was asked to see the patient for safety evaluation and behavioral control.

**Figure 3:**
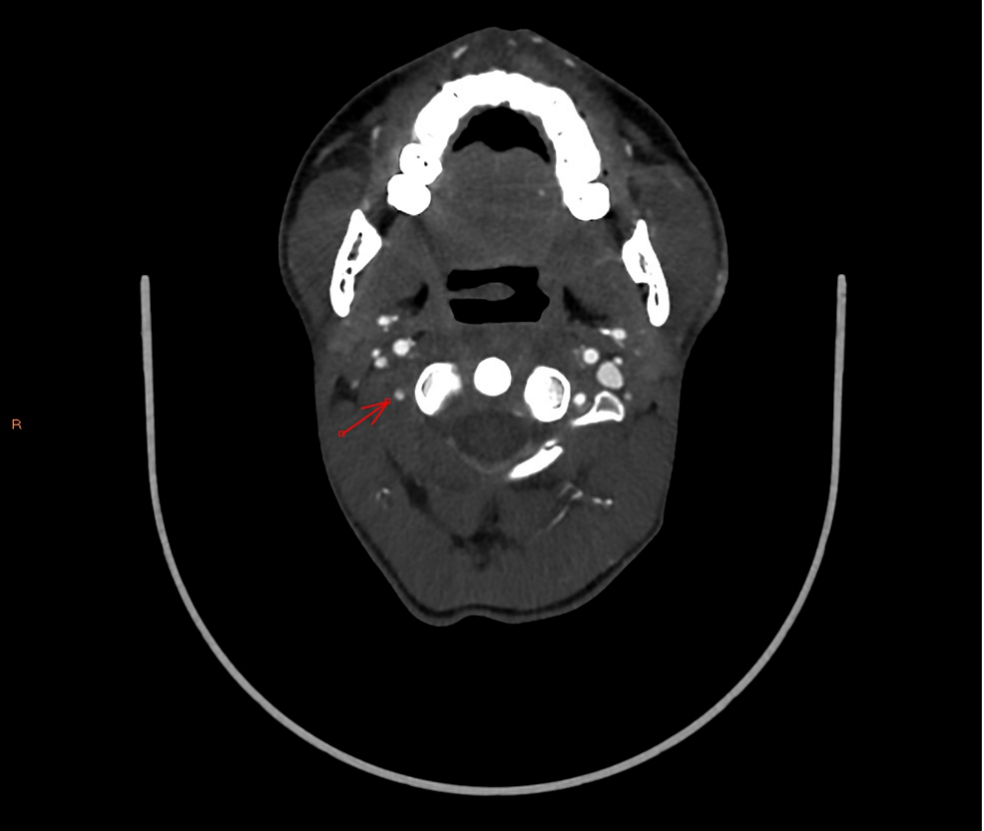
Right vertebral artery (pre-occlusion) - axial image

**Figure 4:**
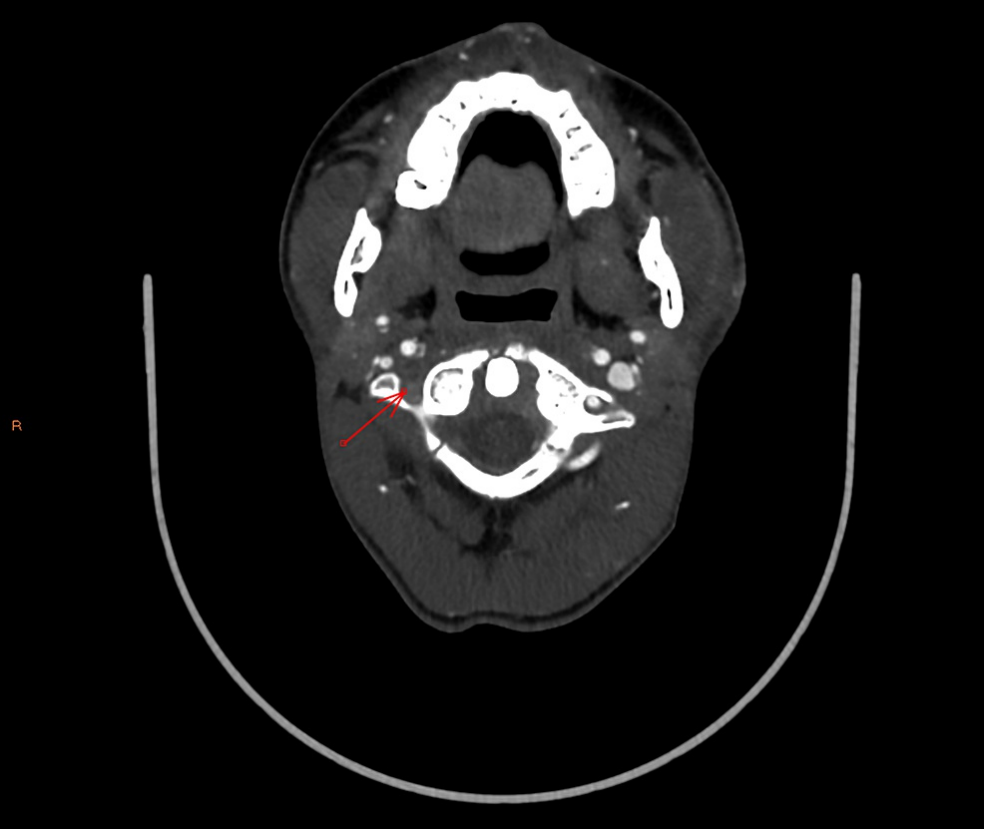
Right vertebral artery (post-occlusion) - axial image

**Figure 5:**
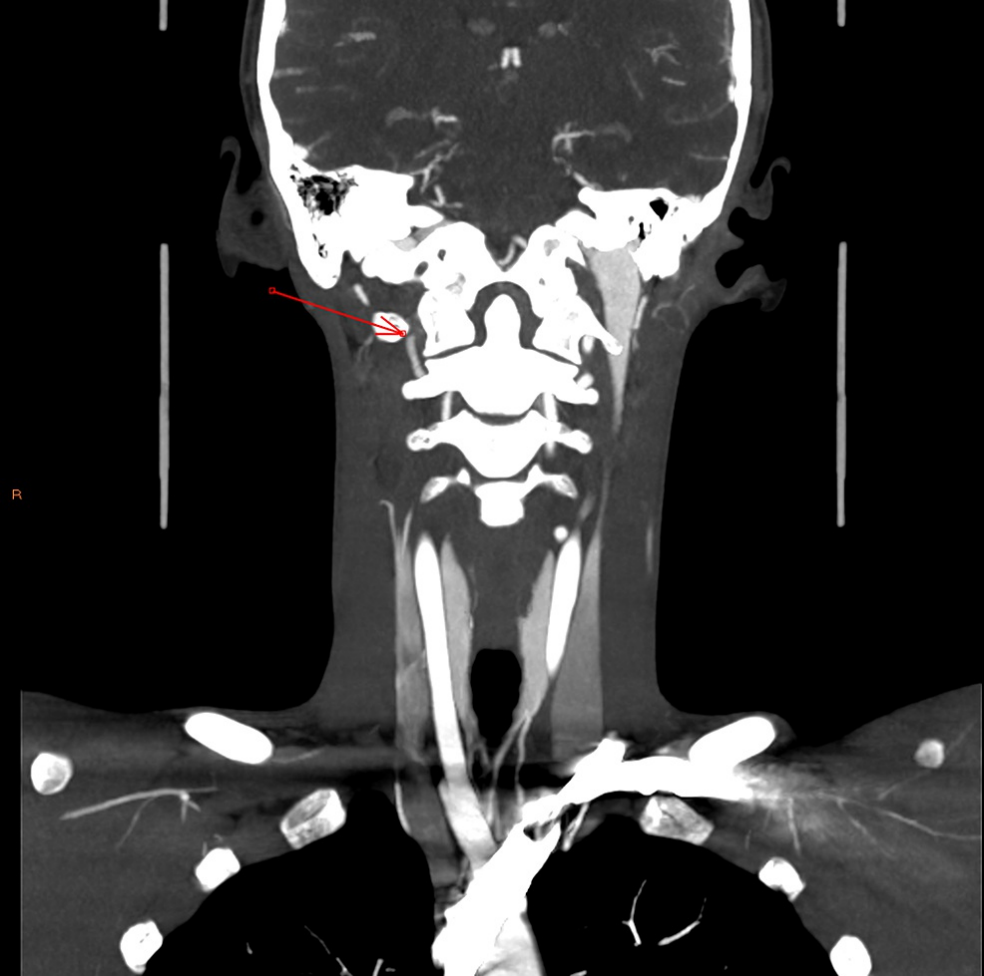
Right vertebral artery occlusion - coronal image

On this admission, CTA revealed a grade IV blunt cerebrovascular injury of the right vertebral artery that had not been present on previous admission (Figures 3-5). Subsequent MRI showed acute and subacute right cerebellar strokes. A hard cervical collar was recommended for prevention of neck manipulation, and a heparin drip was started due to concern for stroke. Neurosurgery expressed strong concern that further neck popping could lead to further vascular injury with subsequent stroke, further neurologic deficits, and possibly death.

Despite repeated education about the potential for worsening neurovascular injuries and her being able to show understanding of these concerns, Ms. M continued to manipulate her neck while in the hospital, resulting in the need for 1:1 constant observation, frequent de-escalation by staff, as-needed medication for severe agitation, and intermittent four-point restraints. On C-L psychiatry evaluation, Ms. M reported that she had been compliant with fluoxetine since her last admission, with an increase in dose to 20 mg daily. Despite this increase, she reported no improvement in anxiety or compulsive neck popping behaviors. She was able to clearly state what the risks were of her continuing this behavior, but it was not clear to the team whether she had a full appreciation for the severity of the potential injury if she continue manipulating her neck. She would comment, “It’s my body! I can do it if I want! I don’t care if I die!” Given her degree of agitation, uncontrolled anxiety, and continued discomfort leading to neck popping, the decision was made to convert the 72-hour involuntary hold to a 90-day involuntary hold for grave disability and admit her to inpatient psychiatry.

On admission, Ms. M had several episodes of increased anxiety and agitation, during which she presented as tearful and easily irritated. Given continued urges to pop her neck, Ms. M was initially placed on 1:1 monitoring, with a stepwise decrease in the level of monitoring as admission progressed. She began to participate in group therapy and engage in family meetings. Fluoxetine was switched to duloxetine to target pain, anxiety, and OCD symptoms. Gabapentin and cyclobenzaprine were given for neck pain with some reported benefits. By day six of hospitalization, Ms. M demonstrated improved insight regarding pain and anxiety, as well as more effective coping skills with the ability to avoid removing her C-collar or popping her neck in the absence of close monitoring. She was no longer felt to be gravely disabled from a psychiatric condition, so the 90-day involuntary hold was discontinued. While the diagnosis of OCD was considered, Ms. M’s Y-BOCS score decreased to 6 (thought to be secondary to improved pain management and coping skills), and discharge diagnoses were somatic symptom disorder, GAD, and MDD. She was instructed to follow up with her local community mental health center, radiology for a follow-up CT scan, and neurosurgery clinic.

Over the course of the following six months, Ms. M continued to present to the ED with compulsive neck popping behavior on a nearly monthly basis, although not requiring medical or psychiatric admissions. A new C7 fracture was also found on a CT scan at an outside hospital (no imaging available to us). On one ER visit, Ms. M noted, “I have these thoughts that haunt me and intrude in my mind, and I can’t dismiss them. The only thing that will make the feeling go away for a moment is to crack my neck and contort my neck in an abnormal position.” She had not followed up with outpatient mental health services and has now been lost to follow-up over the past year.

## Discussion

Although it is known that OCD may involve self-injurious compulsions, there are few cases reported of severe physical injury resulting from these behaviors. The present case is significant because it involves obsessive-compulsive behaviors that directly threaten serious injury including paralysis or even death. However, in order to optimize patient treatment and outcomes, we must first try and elucidate a clear diagnosis.

Ms. M’s compulsive behaviors began at approximately the mean age of onset for OCD. She displays repetitive behavior that she feels driven to perform in response to intrusive thoughts that reduce (albeit temporarily) her anxiety. The compulsions also cause significant distress and physical impairment. Ms. M scored a 28 initially on Y-BOCS. There is physical evidence of excoriation disorder with skin picking that she tries to stop but cannot, which causes her distress. In addition, per collateral from her mother, Ms. M has a history of body-focused repetitive behavior disorder symptoms (e.g., nail biting and lip biting), which caused distress in times of heightened anxiety. In addition, OCD is a condition that may require supratherapeutic doses of psychotropic medications for treatment. Ms. M never received such dosing, and her compulsive neck cracking continued as of the last known clinical contact. For these reasons, we believe Ms. M’s primary psychiatric diagnosis falls on the spectrum of obsessive-compulsive and related disorders.

Another diagnosis to consider is somatic symptom disorder. If popping her neck actually relieved physical discomfort, this may not be entirely consistent with OCD, as compulsions are usually not connected in a realistic way with what they are designed to neutralize or prevent [2]. However, Ms. M had multiple workups for arthralgia, and the causes of neck pain outside of her neck popping behavior (and injury sequelae of this behavior) were not identified. Ms. M also did not respond to standard pain medication regimens that were recommended based on her report of symptoms. Of note, to our knowledge, the patient did not follow up with the rheumatology clinic or physical therapy as was advised. Ms. M certainly had physical symptoms, specifically joint pain and the physical sensation of joints needing to be popped, that had resulted in distress and significant disruption of her daily life, persistently high level of anxiety about the symptoms, and excessive time and energy devoted to these symptoms. Somatic symptom disorder however involves recurrent ideas about somatic symptoms or illness that are less intrusive and without the associated repetitive behaviors aimed at reducing anxiety that occur in obsessive-compulsive disorder [2]. Whether Ms. M’s neck popping was aimed more at reducing anxiety from intrusive thoughts versus reducing perceived pain and discomfort would help clarify the diagnosis. As she has reported both to be true, it is also possible that she may have both diagnoses.

Treatment for OCD involves the management of intrusive thoughts and compulsive behaviors through medication and therapy, such as exposure and response prevention therapy [10]. Somatic symptom disorder management involves regular visits with a trusted provider and the development of coping skills to address physical symptoms [11]. What may be most beneficial for Ms. M then would be to engage in consistent outpatient follow-up to practice these therapies, receive education on physical symptom management, and try optimized doses of psychotropic medication [10,11].

In terms of future treatment directions, it is important to consider acute versus chronic risk of injury if and when considering involuntary treatment. The level of risk of physical harm to Ms. M necessitated involuntary treatment on at least two separate occasions, one leading to a very short psychiatric admission (one day) and one leading to a more typical psychiatric admission length (seven days). After both admissions, there is still a question of what Ms. M’s primary psychiatric diagnosis is, and as of the last known contact, there is still a lack of outpatient mental health follow-up with continued ED visits for her compulsive neck cracking behavior. Ms. M at no point in time ever endorsed purposefully trying to harm herself with these behaviors. The question then is more about whether she is gravely disabled due to a psychiatric condition that then necessitates involuntary psychiatric treatment. Throughout her hospital presentations, she had been able to clearly state what the risks of this ongoing behavior are and what the benefits of the proposed treatment are, and she always self-presents to the ED for help. She has shown a consistent capability of understanding and manipulating information to make informed medical decisions. Now, imagine Ms. M returning in the future with a new physical injury, seeking help, but refusing inpatient psychiatric care. If she has the capacity to make informed medical decisions, albeit ill-advised decisions, then would we as providers be acting paternalistically by demanding involuntarily treatment? Would we be relying on patient autonomy while perhaps uncontrolled anxiety is clouding the patient’s judgment? Would we be illegally holding the patient involuntarily if we could not definitively say that she is gravely disabled by a psychiatric condition?

Cases like this are difficult when patients display the capacity to make informed medical decisions at the moment but then over time do not follow through with the recommended treatment interventions. This can lead to feelings of frustration and helplessness in providers, which can then lead to medical decisions that embrace more paternalism and less autonomy, particularly when significant patient morbidity and/or mortality are the possible outcomes. In Ms. M’s case, her chronic risk for serious or permanent injury is elevated. Clinicians must focus their examination on whether her anxiety is interfering with her ability to rationally process and manipulate information to come to a decision. Even if the ultimate decision is viewed as “unreasonable,” it is the rational processing of information to come to that decision that is paramount [12]. If anxiety does not allow for rational processing of information, then we assert that one must act on the principle of beneficence and consider psychiatrically admitting her involuntarily, even if it is against her wishes. If however Ms. M is able to clearly and rationally explain the process by which she came to her decision to refuse treatment, then we must respect patient autonomy, not violate her civil liberties, and let her discharge, despite the chronically elevated risk of serious morbidity or mortality.

## Conclusions

This case demonstrates the clinical complexities in the diagnosis and treatment of patients with compulsive behaviors that can lead to serious physical harm. Determining an accurate diagnosis has an impact on current and future treatment decisions. Diagnostic clarity is important when communicating what to expect on future visits with the patient, their family, and other medical teams involved in the patient’s care. Providers must consider ethical principles such as beneficence and autonomy when determining the next steps in treatment planning. This is particularly important when providers may feel frustrated and helpless with their role in the patient’s care, which may lead to a subconscious or conscious desire to lean toward more paternalistic interventions at the expense of patient autonomy.
